# Identification of a novel mutation in the *KITLG* gene in a Chinese family with familial progressive hyper- and hypopigmentation

**DOI:** 10.1186/s12920-020-00851-5

**Published:** 2021-01-06

**Authors:** Jianbo Wang, Weisheng Li, Naihui Zhou, Jingliu Liu, Shoumin Zhang, Xueli Li, Zhenlu Li, Ziliang Yang, Miao Sun, Min Li

**Affiliations:** 1 Department of Dermatology, Henan Provincial People’s Hospital, Henan University People’s Hospital, Zhengzhou, 450003 China; 2 Institute for Fetology, The First Affiliated Hospital of Soochow University, Suzhou City, Jiangsu China; 3 Department of Dermatology, The First Affiliated Hospital of Soochow University, Suzhou City, Jiangsu China

**Keywords:** Familial progressive hyper- and hypopigmentation, *KITLG* gene, Mutation, VTNNV motif

## Abstract

**Background:**

Familial progressive hyper- and hypopigmentation (FPHH, MIM 145250) is a rare hereditary skin disorder that is predominantly characterized by progressive, diffuse, partly blotchy hyperpigmented lesions intermingled with scattered hypopigmented spots, lentigines and sometimes Cafe-au-lait spots (CALs). Heterozygous mutations of the KIT ligand (*KITLG*, MIM 184745) gene are responsible for FPHH. To date, only eight *KITLG* mutations have been reported to be associated with FPHH, and no clear genotype–phenotype correlations have been established. This study aimed to identify the causative mutations in the *KITLG* gene in two Chinese FPHH patients.

**Methods:**

Direct sequencing of the coding regions of *KITLG* was performed. Pathogenicity prediction was performed using bioinformatics tools, including SIFT, Polyphen2, and SWISS-MODEL, and the results were further evaluated according to the 2015 American College of Medical Genetics and Genomics (ACMG) guidelines.

**Results:**

The novel mutation c.104A > T (p.Asn35Ile) and the recurrent mutation c.101C > T (p.Thr34Ile) in *KITLG* were identified. As shown using SIFT and Polyphen-2 software, both mutations identified in this study were predicted to be detrimental variations. Three-dimensional protein structure modeling indicated that the mutant KITLG proteins might affect the affinity of KITLG for its receptor, c-KIT. According to the 2015 ACMG guidelines, the novel mutation c.104A > T was ‘likely pathogenic’.

**Conclusions:**

To date, most of the identified *KITLG* mutations have been clustered within the conserved VTNNV motif (amino acids 33–37) in exon 2. The known mutations are only involved in 33 V, 34 T, 36 N, and 37 V but not 35 N. We have now identified a novel mutation in *KITLG*, c.104A > T, that was first reported in FPHH within the conserved 35 N motif. These results strengthen our understanding of FPHH and expand the mutational spectrum of the *KITLG* gene.

## Background

Familial progressive hyper- and hypopigmentation (FPHH, MIM 145250) is a rare genetic skin pigmentation anomaly disorder characterized by progressive, diffuse, partly blotchy hyperpigmented lesions that are intermixed with multiple café-au-lait spots, hypopigmented maculae and lentigines that are located on the face, neck, trunk and limbs and frequently on the palms, soles and oral mucosa. Dispigmentation patterns can range from well-isolated café-au-lait/hypopigmented patches on a background of normal-appearing skin to a confetti-like or mottled appearance [1, 2].

The FPHH locus was mapped at chromosome 12q21.31-q23.1 by genome-wide linkage analysis in a six-generation Chinese family. Positional candidate gene screening revealed that a heterozygous transversion (c.107A > G; p.Asn36Ser) in exon 2 of the KIT ligand (*KITLG*, MIM 184745) gene is responsible for this disorder [3].

The *KITLG* gene, also known as stem cell factor (SCF) and mast cell growth factor, encodes the ligand for the KIT receptor tyrosine kinase. By KITLG binding, KIT dimerizes and initiates diverse cellular responses and plays a crucial role in the development and maintenance of the melanocyte lineage in adult skin. Injection of the soluble form of sKITLG resulted in hyperpigmentation of the grafted skin tissue, while injection of the KIT- or KITLG-blocking antibodies into the explanted human skin led to a loss of melanocytes [4]. Functional analysis of the soluble form of KITLG (sKITLG) showed that mutant sKITLG (Asn36Ser) increased the content of melanin by 109% compared with wild-type sKITLG in human melanoma cells, and a gain-of-function effect of this missense mutation was indicated to possibly trigger hyperpigmentation of skin in patients [3].

Several mutations (HGMD, http://www.hgmd.cf.ac.uk) in the *KITLG* gene have been documented in a few FPHH families [2, 5–7]. Most of the FPHH-causing mutations in *KITLG* are clustered within the conserved VTNNV motif (amino acids 33–37) in exon 2, and a mutated VTNNV domain may increase the affinity of KITLG to the c-Kit receptor, suggesting that the mutation causes a downstream gain-of-function effect. However, many FPHH families without *KITLG* mutations have been identified, indicating additional locus heterogeneity for this disorder [5, 8, 9].

Here, we reported two Chinese progressive hyper- and hypopigmentation cases, with one being familial and the other being sporadic. A novel mutation c.104A > T (p.Asn35Ile) and a recurrent mutation c.101C > T (p.Thr34Ile) in the *KITLG* gene were identified. Furthermore, we summarized the information on the mutations of the *KITLG* gene associated with the progressive hyper- and hypopigmentation previously reported.

## Methods

### Characteristics of the participants

**Fig. 1:**
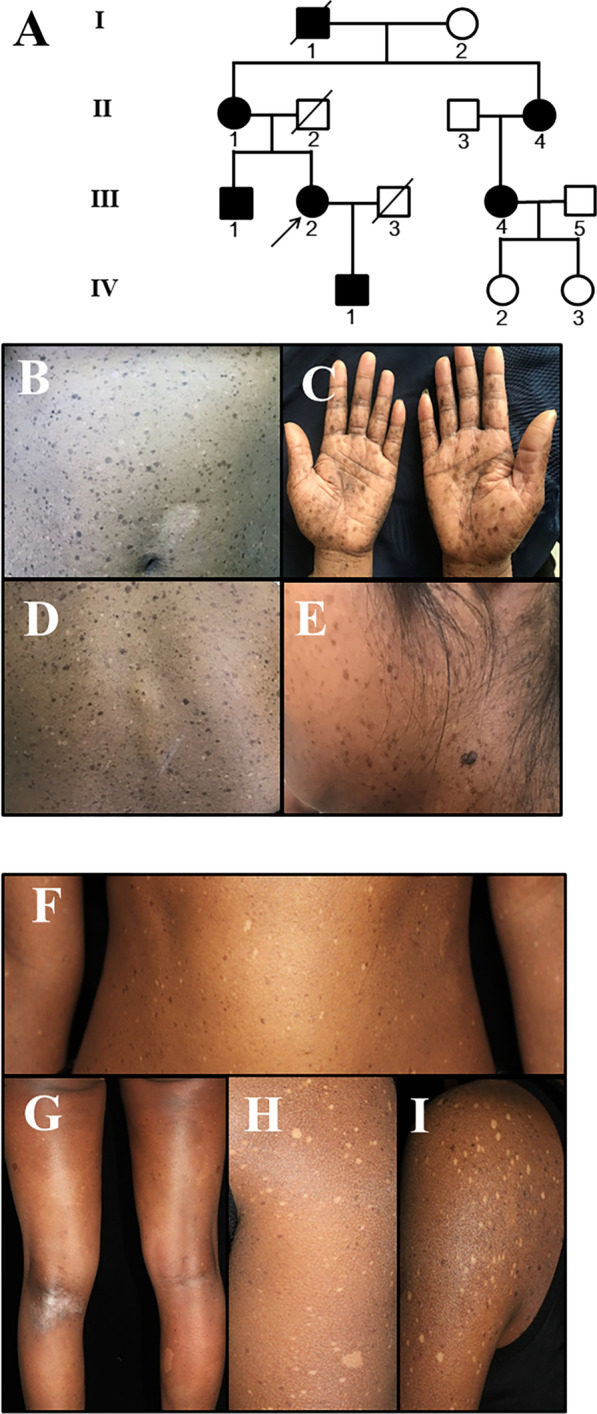
Pedigree and clinical features of the two cases with familial progressive hyper- and hypopigmentation. **a** Pedigree of family 1. **b**–**e** Generalized hyper- and hypopigmentation with irregular patches was found at birth, and the patches (0.2–0.8 cm) progressed successively over her trunk, limbs, face and neck with age. **f**–**i** With age, the lesions increased in both size and number and became more noticeable and appeared on the trunk and limbs

Family 1 was a four-generation Chinese FPHH pedigree with seven affected individuals (three men and four women) (Fig. 1a; Additional file 1: Fig. S1). The pedigree presented an autosomal dominant inheritance pattern (Fig. 1a). The proband (III2) from family 1 was a 37-year-old woman. Generalized hyper- and hypopigmentation with irregular patches was found at birth, and the patches (0.2–0.8 cm) progressed successively over her face, neck, trunk and limbs with age. There were also a small number of larger pigmented lesions that were several centimeters in diameter on her trunk and limbs (Fig. 1b–e). All the affected individuals in this family had similar lesions, and none of them showed any other skin, nail, hair, teeth, mucosal or systemic diseases.

The sporadic case was a 26-year-old woman, and her parents were unaffected (Additional file 2: Fig. S2). The clinical appearances of this sporadic patient were mostly similar to those seen in the proband of family 1. However, diffuse hyperpigmentation was found on her entire body, and two vast café-au-lait (CAL)-like lesions presented on her legs at birth. One week after birth, it was shown that her diffuse hyperpigmented skin was intermixed with some small lentigines/CAL-like lesions, as well as hypopigmented macules and spots on her trunk. With increasing age, the lesions increased in both size and number and became more noticeable and appeared on her trunk and limbs (Fig. 1f–i). She was born to healthy, nonconsanguineous parents. Her nails, hair, teeth and mucosae were normal.

### Mutation screening of the *KITLG* gene by direct sequencing

Genomic DNA was extracted from blood samples of family 1 and a sporadic case using the QIA-amp^®^ DNA Blood Mini Kit (Qiagen, Shanghai, China). All exons and their flanking intronic sequences of the *KITLG* gene were amplified by polymerase chain reaction (PCR) as described previously [8]. Purified PCR products were sequenced directly using an ABI Prism^®^ 3730 automated sequencer (Applied Biosystems, Foster City, CA, USA). DNA sequences were analyzed by comparison to the human *KITLG* reference sequence (NM_000899.5, https://www.ncbi.nlm.nih.gov/nuccore/NM_000899). The mutations were checked with HGMD, Clinvar (https://www.ncbi.nlm.nih.gov/clinvar/) and the 1000 Genomes project (http://www.ncbi.nlm.nih.gov/variation/tools/1000genomes/). Furthermore, samples from 100 unrelated normal Chinese Han individuals were also sequenced to exclude polymorphic variants.

### KITLG protein function prediction and molecular modeling

Online in silico programs were applied to predict the potential impact of an amino acid substitution on the structure and function of the KITLG protein with Polyphen-2 (http://genetics.bwh.harvard.edu/pph2/) and SIFT (http://sift.jcvi.org/), respectively. Furthermore, we performed analysis using an online server, SWISS-MODEL (http://swissmodel.expasy.org/), to construct the three-dimensional structure of KITLG*.* The pathogenicity prediction was evaluated following the American College of Medical Genetics and Genomics (ACMG) guidelines of 2015.

## Results

### Identification of *KITLG* gene mutations

**Fig. 2:**
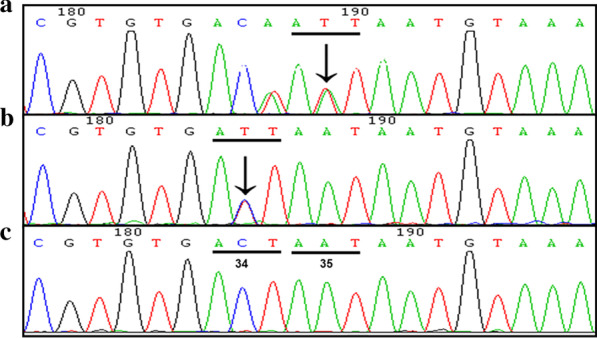
*KITLG* gene mutation analysis in two Chinese FPHH cases. **a** A novel mutation c.104A > T (p.Asn35Ile) was identified in family 1. The variation c.102 T > A (p.Thr34Thr) is a synonymous mutation. **b** A recurrent mutation c.101C > T (p.Thr34Ile) was identified in the sporadic case. **c** Sequence of the wild-type allele showing translation of the threonine residue at codon 34 (ACT) and asparagine at codon 35 (AAT)

All subsequently detected variants were then filtered on the basis of population. After filtering all variants, a novel heterozygous missense mutation c.104A > T (p.Asn35Ile) and a recurrent mutation c.101C > T (p.Thr34Ile) in the *KITLG* gene were revealed in family 1 (Fig. 2a) and in the sporadic case (Fig. 2b). These two mutations were not detected in the unaffected family members or 100 unrelated population-matched controls (Fig. 2c). The variation c.102 T > A (p.Thr34Thr) in family 1 is a synonymous mutation (Fig. 2a).

According to the 2015 ACMG guidelines, the prevalence of the variant in affected individuals was significantly increased compared with the prevalence in controls, meeting the criterion of pathogenic strong 4 (PS4). These two mutations match the criterion of pathogenic moderate 1 (PM1) since all of them are in hot spot regions. A novel heterozygous missense mutation c.104A > T (p.Asn35Ile) was cosegregated in each affected family member, indicating that they matched the criterion of pathogenic supporting 2 (PP2).

### Prediction of the potential impacts of the mutations

The mutations c.104A > T (p.Asn35Ile) and c.101C > T (p.Thr34Ile) were predicted to be ‘possible damaging’ and ‘deleterious’ with Polyphen-2 (http://genetics.bwh.harvard.edu/pph2/) and SIFT (http://sift.jcvi.org/), respectively. These predictions indicated that the two variants may have an effect on protein function, meeting the criterion of pathogenic supporting 3 (PP3) according to the ACMG guidelines of 2015.

**Fig. 3:**
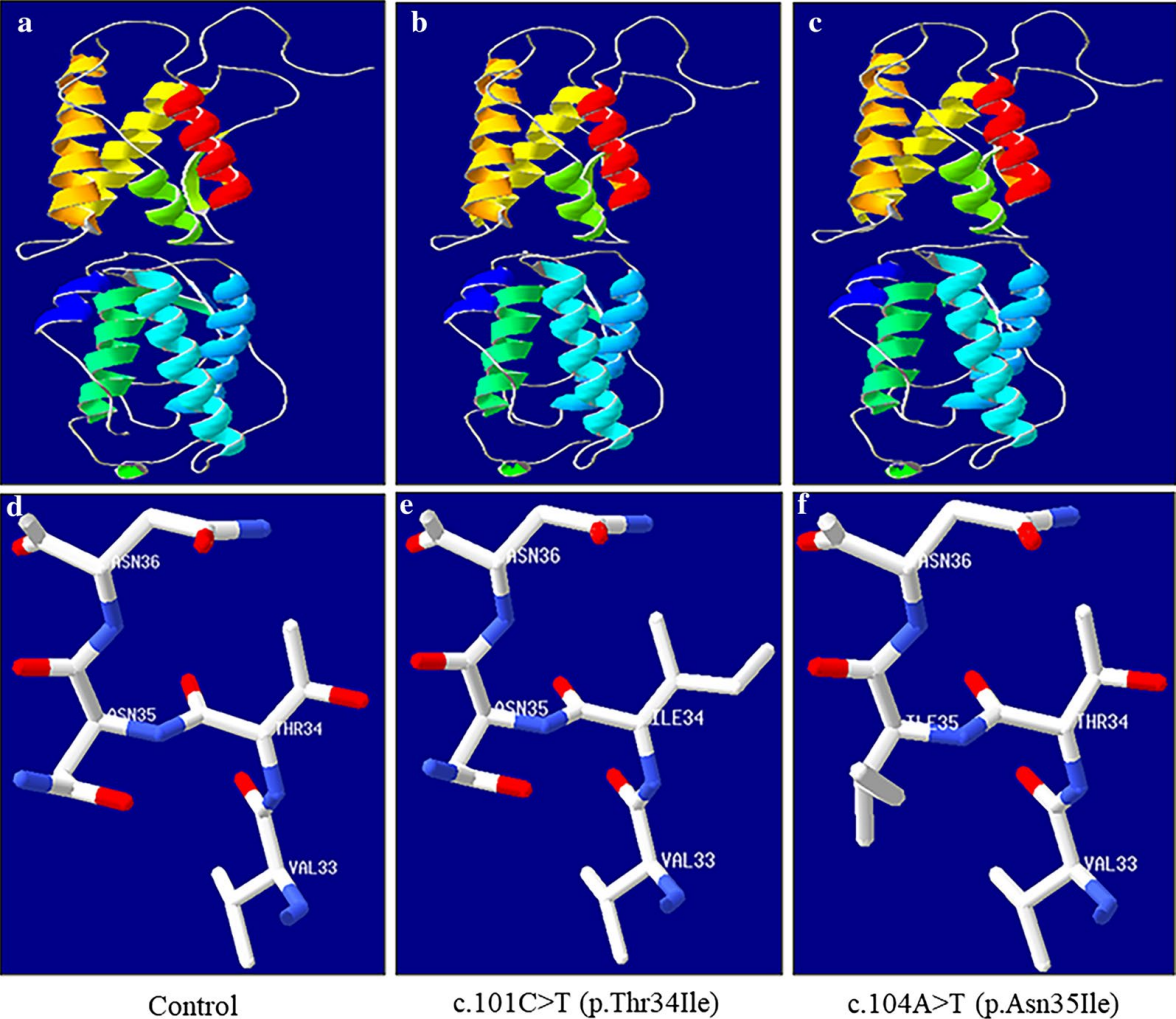
Three-dimensional structure of *KITLG*. **a**–**c** Protein three-dimensional structure overall picture. **d**–**f** Partial map of protein three-dimensional structure. **e** Thr and Asn, as polar, neutral R-based amino acids, became Ile, nonpolar, hydrophobic R-based amino acids. When Thr changed to Ile at position 34, the side chain of Ile 34 changed. **f** When Asn changed to Ile at position 35, the side chain of Ile 35 changed

The substitutions 34Thr and 35Asn were identified in KITLG (Fig. 3), in which polar, neutral, hydrophilic R-based amino acids were changed to Ile, a nonpolar, hydrophobic R-based amino acid, in family 1 and the sporadic case with SWISS-MODEL (http://swissmodel.expasy.org/ ).

Finally, a novel heterozygous missense mutation c.104A > T (p.Asn35Ile) and a recurrent mutation c.101C > T (p.Thr34Ile) in the *KITLG* gene were revealed in family 1 (Fig. 2a) and in the sporadic case and were classified as ‘likely pathogenic’ in accordance with the 2015 ACMG guidelines. The details of each mutation are shown in Additional file 3: Table S1, and the genotype information is provided in Additional file 4: Table S2.

## Discussion

FPHH is a rare autosomal dominant disorder with variable penetrance caused by mutations in the *KITLG* gene, which encodes the C-Kit ligand [5]. Because FPHH is very rare with reduced penetrance, no clear incidence rate of this disease has been documented. Patients with FPHH often do not have systemic symptoms. Growth retardation and intellectual disability in some affected members were reported by Westerhof et al. [10]. A 2-year-old Chinese female FPHH patient with frequent seizures, impaired temperature regulation and susceptibility to infection also had mild intellectual disability [11].

Nine publications in PubMed (https://www.ncbi.nlm.nih.gov/pubmed) were related to pathogenic mutations of the *KITLG* gene. Mutations in the *KITLG* gene are associated with autosomal dominant nonsyndromic deafness-69 (DFNA69, MIM 616697), Waardenburg syndrome-2 (WS2, MIM 193510), and FPHH. Seco Z et al. [12] reported that certain mutations of *KITLG*, including c.286_303delinsT (p.Ser96Ter), c.200_202del (p.His67_Cys68delinsArg), and c.310C > G (p.Leu104Val), cause asymmetric and unilateral hearing loss and Waardenburg syndrome type 2 (WS2). Ogawa Y et al. [13] reported a patient with WS2 who had the unusual complication of large, pigmented macules caused by a homozygous *KITLG* mutation (c.94G > A, p.Arg32Cys). It was speculated that the mechanism of the mutation underlying WS2 leading to membrane incorporation and reducing secretion of KITLG occurs via a gain-of-function or dominant-negative effect. A de novo mosaic *KITLG* variant (NM_000899.3:c.329A > G; p.Asp110Gly) was found in a 6-year-old boy who had congenital linear and mottled hyperpigmentation [14]. However, all the phenotypes presented in these three publications with *KITLG* are not defined as clearly associated with FPHH; therefore, we only summarize all the other *KITLG* mutations associated with FPHH here in this study.

**Fig. 4:**
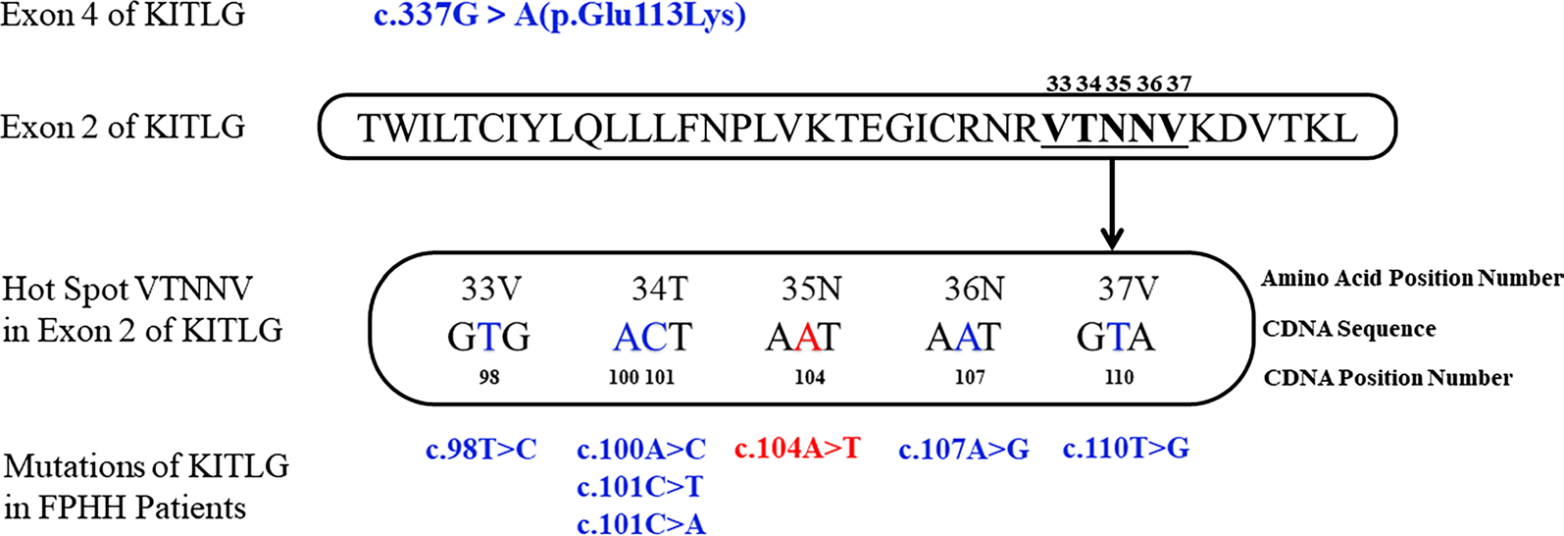
Summary of *KITLG* gene mutations associated with FPHH. All the mutations associated with FPHH are located within the VTNNV region in exon 2, except c.337G > A in exon 4 of the *KITLG* gene. The mutations reported previously are in BLUE, while the novel mutation (c.104A > T) at 35 N reported in this study is in RED

Including the novel mutation (c.104A > T, p.Asn35Ile) we reported in this study, eight different missense mutations in the *KITLG* gene responsible for FPHH have been identified (Additional file 5: Table S3, Fig. 4). Seven out of eight mutations were clustered in a short amino acid sequence (VTNNV, amino acids 33–37) in exon 2 (Fig. 4), except for c.337G > A, which was in exon 4 of the *KITLG* gene. Most pathogenic mutations in FPHH occur within the VTNNV domain of the KITLG protein (amino acids 33–37) and lie within the third b-strand of the protein. Only the p.Val37 change represents the first amino acid of the second a-helix (amino acids 37–46). To date, the reported mutations are only involved in 33 V, 34 T, 36 N, and 37 V but not 35 N. We are the first to report the c.104A > T (p.Asn35Ile) mutation at 35 N (Fig. 4) with FPHH patients.

Except for diffuse hyper- and hypopigmentation, vast CAL-like lesions have been detected as the most common skin problems present in FPHH patients. Vitiligo was found in one family. Sparse lateral eyebrows and malignancy (pharyngeal cancer, papillary thyroid cancer and melanoma) were found in two families. Short sutures were found in only one family, and mental retardation was not present in these FPHH patients (Additional file 5: Table S3).

KITLG, as KIT LIGAND, is produced locally in human skin by epidermal keratinocytes and endothelial cells, where it induces the migration, development and survival of melanocytes. The signaling of *KITLG* and its receptor KIT plays an important role in melanocyte proliferation and pigment production [15, 16]. The role of the KIT/KITLG system in melanogenesis has been experimentally confirmed using animal models [3, 17]. After KITLG binds the c-KIT receptor, dimerization is triggered. It initiates signal transduction via the RAS/MAPK pathway to upregulate melanoblast proliferation [16, 18]. The KITLG/C-KIT/RAS/MAPK signaling pathways play important roles in the regulation of hematopoiesis, stem cell survival, gametogenesis, mast cell development, migration and function, and skin color [19, 20]. Mutant alleles of the *KITLG* gene are lethal in homozygous mice and produce a variable level of coat-color dilution in heterozygous mice [20]. It has been reported in human studies that variations in the *KITLG* gene are also associated with skin, hair, and eye pigmentation (MIM 611664), autosomal dominant nonsyndromic deafness-69, WS2 and FPHH.

Here, we reported a novel c.104A > T (p.Asn35Ile) mutation of *KITLG* in a Chinese FPHH family. According to the 2015 ACMG guidelines, the mutation was initially identified as a ‘likely pathogenic’ mutation [21]. To the best of our knowledge, only eight different missense *KITLG* mutations have been reported to cause FPHH (Additional file 5: Table S3). Notably, seven known mutations were clustered in a highly conserved short amino acid sequence VTNNV (amino acids 33–37) (Fig. 4). The VTNNV domain of the KITLG protein (amino acids 33–37) lies within the third b-strand of the protein and is responsible for its binding functions. Both mutations c.104A > T (p.Asn35Ile) and c.101C > T (p.Thr34Ile) found in this study were located in the VTNNV domain and were predicted to be detrimental variations by the SIFT and Polyphen-2 tools. Using the Swiss-Model servers [22, 23], three-dimensional structures of mutant *KITLG* proteins were found to change compared with the wild type (Fig. 3). Both 35Asn and 34Thr are polar, hydrophilic amino acids, and the mutant became nonpolar, hydrophobic isoleucine; therefore, it might change the features of the protein and affect the ligand affinity to its receptor c-Kit, thus affecting the migration of melanoblasts, melanosome transfer and melanin synthesis, conferring a phenotype with hyper- and hypopigmentation. The precise mechanisms need to be elucidated by further experiments. Our findings revealed a novel *KITLG* mutation associated with FPHH and reinforced the evidence that VTNNV was a hot spot for mutation. However, definitive functional analyses of this mutation are needed to determine the structure–function relationship in patients with FPHH.

## Conclusions

In summary, a novel mutation c.104A > T (p.Asn35Ile) in the *KITLG* gene was reported in a Chinese FPHH patient. The correlations between genotypes and phenotypes of FPHH were summarized. These results strengthen our understanding of FPHH and expand the mutational spectrum of the *KITLG* gene.

## Supplementary Information

**Additional file 1. Fig. S1**: Sequencing results of seven individuals from family 1. A: II1, B: II4, C: III1, D: III4, E: III5, F: IV1, G: IV3

**Additional file 2. Fig. S2**: Sequencing results of the proband’s parents from the sporadic case. A: Proband’s father, B: Proband’s mother.

**Additional file 3. Table S1**: Classification of KITLG mutations in this study according to the 2015 ACMG guidelines.

**Additional file 4. Table S2**: Family 1 (A) and the sporadic case (B) genotype information details.

**Additional file 5. Table S3**: The reported cases and KITLG mutations of familial progressive hyper- and hypopigmentation to date.

## Data Availability

The datasets generated during the current study are available in the Mendeley repository (10.17632/yftvffrkyt.1).

## Abbreviations

FPHH: Familial progressive hyper- and hypopigmentation
CALS: Café-au-lait spots
sKITLG: The soluble form of KITLG
MIM: Mendelian Inheritance in Man
