# Supplementary figures and images for: Identification of a novel mutation in the KITLG gene in a Chinese family with familial progressive hyper- and hypopigmentation
Source: BMC Med Genomics. 2021 Jan 6;14:12. doi: 10.1186/s12920-020-00851-5 (PMC7789533; doi:10.1186/s12920-020-00851-5)

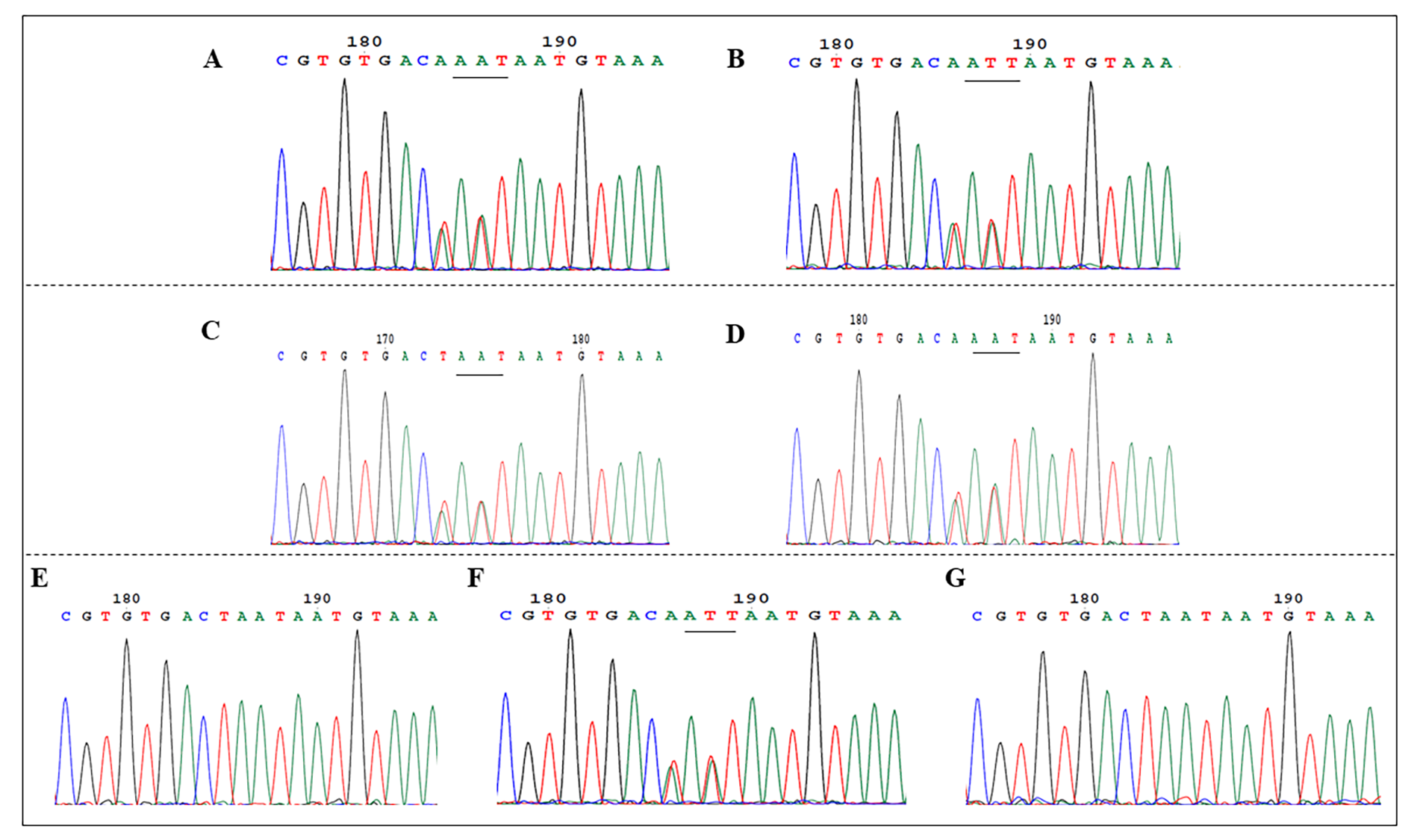

Supplement: Supplementary file 1 — Additional file 1. Fig. S1: Sequencing results of seven individuals from family 1. A: II1, B: II4, C: III1, D: III4, E: III5, F: IV1, G: IV3 [file 12920_2020_851_MOESM1_ESM.tif]

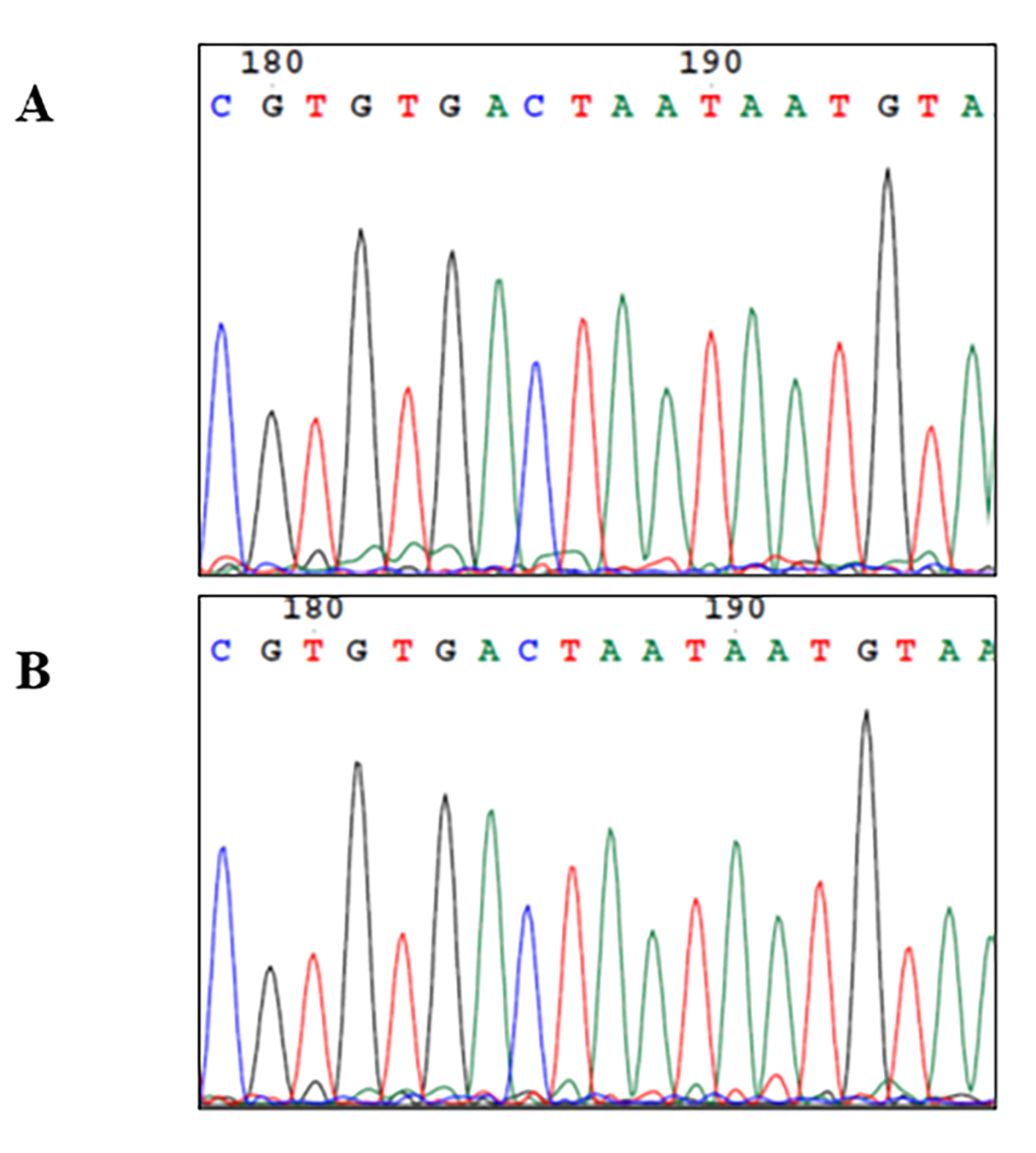

Supplement: Supplementary file 2 — Additional file 2. Fig. S2: Sequencing results of the proband’s parents from the sporadic case. A: Proband’s father, B: Proband’s mother. [file 12920_2020_851_MOESM2_ESM.tif]
