# Supplementary material for: Identification of a novel mutation in the KITLG gene in a Chinese family with familial progressive hyper- and hypopigmentation
Source: BMC Med Genomics. 2021 Jan 6;14:12. doi: 10.1186/s12920-020-00851-5 (PMC7789533; doi:10.1186/s12920-020-00851-5)
Supplement: Supplementary file 3 — Additional file 3. Table S1: Classification of KITLG mutations in this study according to the 2015 ACMG guidelines. [file 12920_2020_851_MOESM3_ESM.docx]

**Table1**. Classification of *KITLG* mutations in this study according to the ACMG guildline

| Family ID | Inheritance | Variant | | | | PS4 | PM | PP | | | Classification |
| --- | --- | --- | --- | --- | --- | --- | --- | --- | --- | --- | --- |
|  |  | cDNA change | p.change | status | type | PS4 | PM1 |  | PP2 | PP3 |  |
| Family1 | AD | c.104A>T | p.Asn35Ile | Hetero | missense | Y | Y |  | Y | Y | Likely Pathogenic |
| AD autosomal dominant, Hetero heterozygosity | | | | | | | | | | | |
| PS4 = The prevalence of the variant in affected individuals is significantly increased compared with the prevalence in controls; | | | | | | | | | | | |
| PM1 = Located in a mutational hot spot and/or critical and well-established functional domain (e.g., active site of an enzyme) without benign variation; | | | | | | | | | | | |
| PP2 = Missense variant in a gene that has a low rate of benign missense variation and in which missense variants are a common mechanism of disease | | | | | | | | | | | |
| PP3 = Multiple lines of computational evidence support a deleterious effect on the gene or gene product (conservation, evolutionary, splicing impact, etc.) | | | | | | | | | | | |
