# Supplementary material for: Identification of a novel mutation in the KITLG gene in a Chinese family with familial progressive hyper- and hypopigmentation
Source: BMC Med Genomics. 2021 Jan 6;14:12. doi: 10.1186/s12920-020-00851-5 (PMC7789533; doi:10.1186/s12920-020-00851-5)
Supplement: Supplementary file 4 — Additional file 4. Table S2: Family 1 (A) and the sporadic case (B) genotype information details. [file 12920_2020_851_MOESM4_ESM.docx]

**Table 2**. The *KITLG* mutation of family 1 (A) and the sporadic case (B)

**A**

| **ID** | **Genotype** |
| --- | --- |
| II1 | +/- |
| II4 | +/- |
| III1 | +/- |
| Proband (III2) | +/- |
| III4 | +/- |
| III5 | +/+ |
| IV1 | +/- |
| IV3 | +/+ |

+/+: Wild type; +/-: Heterozygous mutation c.104A>T.

**B**

| **ID** | **Genotype** |
| --- | --- |
| Proband’s father | +/+ |
| Proband’s mother | +/+ |
| Proband | +/- |

+/+: Wild type; +/-: Heterozygous mutation c.101C>T.
