# Supplementary material for: Identification of a novel mutation in the KITLG gene in a Chinese family with familial progressive hyper- and hypopigmentation
Source: BMC Med Genomics. 2021 Jan 6;14:12. doi: 10.1186/s12920-020-00851-5 (PMC7789533; doi:10.1186/s12920-020-00851-5)
Supplement: Supplementary file 5 — Additional file 5. Table S3: The reported cases and KITLG mutations of familial progressive hyper- and hypopigmentation to date. [file 12920_2020_851_MOESM5_ESM.docx]

| **Table 3.** The reported cases and *KITLG* mutations of familial progressive hyper- and hypopigmentation to date | Reference | [1] | [2,3] | [2,3] | [2,4] | [2] | [5] | [5] | [6] | [7] | [8] | This study | This study |
| --- | --- | --- | --- | --- | --- | --- | --- | --- | --- | --- | --- | --- | --- |
|  | Amino acid changes | p.Asn36Ser | p.Asn36Ser | p.Asn36Ser | p.Val33Ala | p.Thr34Pro | p.Thr34Asn | p.Val37Gly | p.Thr34Ile | p.Thr34Ile | p.Glu113Lys | p.Thr34Ile | p.Asn35Ile |
|  | Variant type | Missense | Missense | Missense | Missense | Missense | Missense | Missense | Missense | Missense | Missense | Missense | Missense |
|  | Mutation | c.107A>G | c.107A>G | c.107A>G | c.98T>C | c.100A>C | c.101C>A | c.110T>G | c.101C>T | c.101C>T | c.337G＞A | c.101C>T | c.104A>T |
|  | Exon | 2 | 2 | 2 | 2 | 2 | 2 | 2 | 2 | 2 | 4 | 2 | 2 |
|  | Cancer |  |  |  |  |  | pharyngeal cancer; melanoma | papillary thyroid cancer |  |  |  |  |  |
|  | Height |  |  |  |  |  | below average |  |  |  |  |  |  |
|  | Clinical manifestation | diffuse hyperpigmentation | diffuse hyperpigmentation, hypopigmented ash-leaf-like lesions, cafe´-au-lait macules, lentigines | diffuse hyperpigmentation, confetti-like hypopigmented macules, cafe´-au-lait macules, lentigines | hyper- and hypopigmented macules/patches, cafe´-au-lait macules | diffuse hyperpigmentation, with scattered cafe´-au-lait macules, lentigines, small hypopigmented spots and vitiligo | hyper- and hypopigmented macules, cafe´-au-lait-like macules and large irregular hypopigmented macules, unusually sparse lateral eyebrows | generalized freckling and hyperpigmentation, lipo-oedema of the legs, unusually sparse lateral eyebrows | diffuse hyperpigmentation intermixed with CALS, lentigines, hypopigmented macules and spots | diffuse hyperpigmentation with irregular café-au-lait and café noir spots, ash leaf-like macules, hypopigmented patches and intense hyperpigmentation | irregularly shaped asymptomatic hyper- and hypopigmented macules | diffuse hyper- hypopigmentation and vast café-au-lait like lesions | generalized hyper- and hypopigmentation |
|  | Nationality | Chinese | Germany | Germany | French Canadian origin | Danish | British | British | Chinese | Turkey | Japanese | Chinese | Chinese |
|  | Patients | Family 1 | Family 2 | Family 3 | Family 4 | Family 5 | Family 6 | Family 7 | Sporadic case 1 | Sporadic case 2 | Family 8 | Sporadic case 3 | Family 9 |

**Table 3 References:**

1. Wang ZQ, Si L, Tang Q, Lin D, *et al*. Gain-of-function mutation of KIT ligand on melanin synthesis causes familial progressive hyperpigmentation. Am J Hum Genet. 2009;84(5):672-7.

2. Amyere M, Vogt T, Hoo J, et al. KITLG mutations cause familial progressive hyper- and hypopigmentation. J Invest Dermatol. 2011;131(6):1234-9.

3. Zanardo L, Stolz W, Schmitz G, Kaminski W, Vikkula M, Landthaler M, Vogt T. Progressive hyperpigmentation and generalized lentiginosis without associated systemic symptoms: a rare hereditary pigmentation disorder in south-east Germany. Acta Derm Venereol. 2004;84(1):57-60.

4. Hoo JJ, Shrimpton AE. Familial hyper- and hypopigmentation with age-related pattern change. Am J Med Genet A. 2005;132A(2):215-8.

5. Cuell A, Bansal N, Cole T, Thind CK *et al*. Familial progressive hyper- and hypopigmentation and malignancy in two families with new mutations in KITLG. Clin Exp Dermatol. 2015;40(8):860-4.

6. Zhang J, Cheng R, Liang J, Ni C, Li M, Yao Z. Report of a child with sporadic familial progressive hyper- and hypopigmentation caused by a novel KITLG mutation. Br J Dermatol. 2016;175(6):1369-71.

7. Gulseren D, Guleray N, Akgun-Dogan O, Simsek-Kiper PO, Utine EG, Alikasifoglu M, Ersoy-Evans S. Cafe noir spots: a feature of familial progressive hyper- and hypopigmentation. J Eur Acad Dermatol Venereol. 2020;34(2):e76-e77.

8. Kato M, Yagami A, Tsukamoto T, Shinkai Y, Kato T, Kurahashi H. Novel mutation in the KITLG gene in familial progressive hyperpigmentation with or without hypopigmentation. J Dermatol. 2020;
